# Supplementary material for: Logarithmically scaled, gamma distributed neuronal spiking
Source: J Physiol. 2022 Oct 6;601(15):3055–69. doi: 10.1113/JP282758 (PMC10952267; doi:10.1113/JP282758)
Supplement: Supplementary file 1 — Statistical Summary Document [file TJP-601-3055-s002.docx]

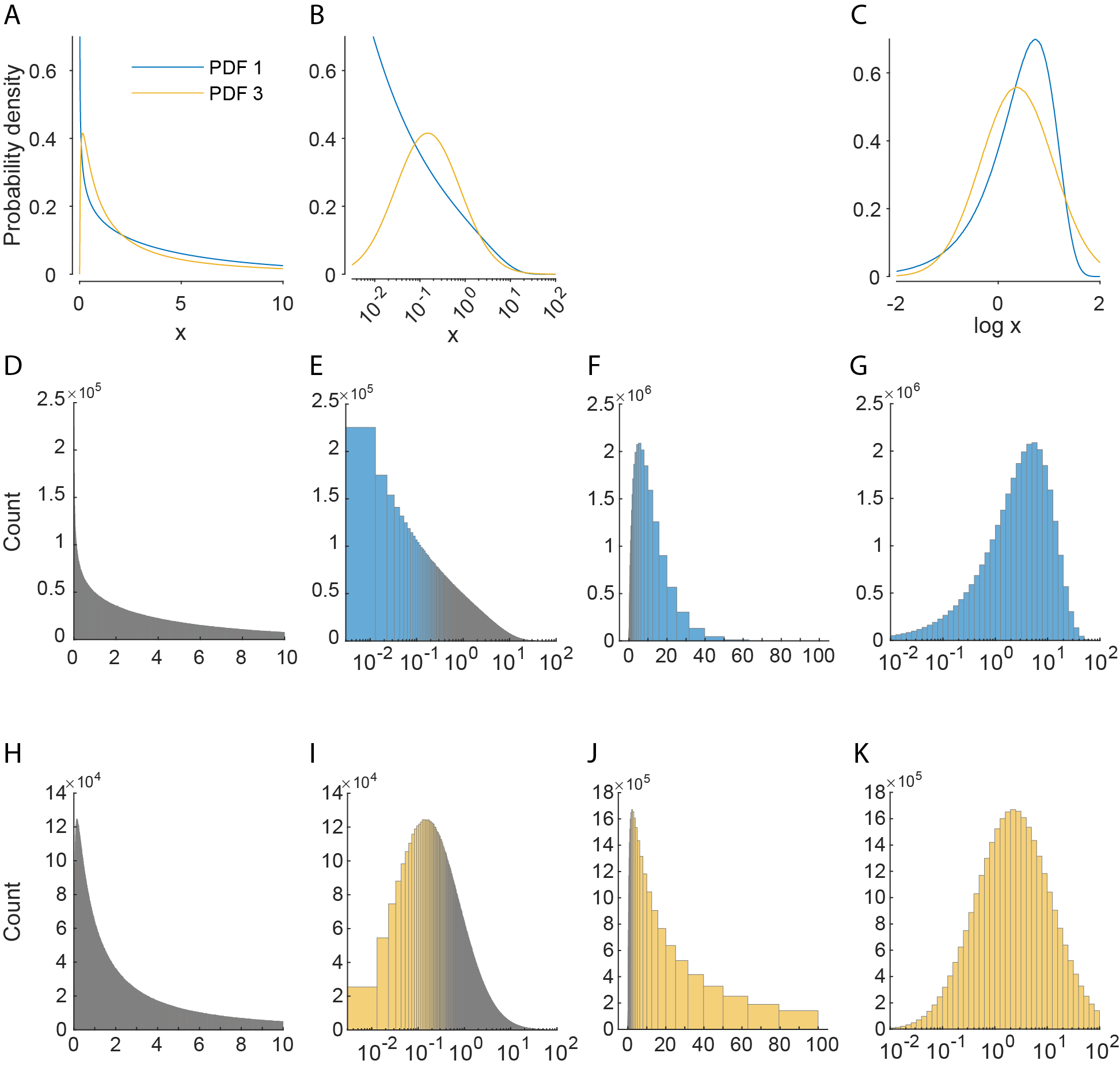


**(A-C)** An example gamma PDF (PDF 1, blue) and an example lognormal PDF (PDF 3, orange), shown in the same format as Fig. 2 of the main article. **(D)** Histogram of data distributed according to PDF 1: 3×10^7^ data points, bin width of 0.01. **(E)** Same as D, with x-axis logarithmically (rather than linearly) scaled. **(F)** Histogram of the same data points using bins whose size is not uniform (as in D-E) but rather exponential in each bin’s location on the x-axis. **(G)** Same as F, with x-axis logarithmically scaled. This is equivaled to a PDF (and a conventional histogram) of log-transformed data. In D-G, bins have grey outlines. **(H-K)** Same format as D-G, for 3×10^7^ data points distributed according to PDF 3.

MATLAB code for generating this figure is publicly available at
<github.com/m-okun/LogSpiking_JPhysiol>
